# Children’s Health: The Opposite of Obesity: Undernutrition Overwhelms the World’s Children

**DOI:** 10.1289/ehp.112-a802

**Published:** 2004-10

**Authors:** Carol Potera

An alarming number of studies report that overnutrition and the resulting obesity are a growing health problem for children in industrialized nations and even some developing ones. The explosion of such studies might seem to suggest that starvation is a thing of the past, yet children in many developing countries still go hungry. Furthermore, a lack of calories and nutrients—or undernutrition—can worsen the effects of infectious disease, and thereby causes half of all child deaths worldwide, report public health experts at The Johns Hopkins University and the World Health Organization in the 1 July 2004 issue of the *American Journal of Clinical Nutrition*.

This new finding supports a 1995 study coordinated by David Pelletier, an associate professor of nutrition sciences at Cornell University, which provided the first evidence of how often child deaths are attributable to under-nutrition. The latest study goes a step further: Johns Hopkins nutritionist Laura Caulfield and her colleagues answer the important question of whether undernutrition exacerbates the effects of infectious diseases.

Caulfield headed a team that analyzed data from 10 large studies of child deaths in sub-Saharan Africa and Southeast Asia. These studies included data about the average weight-for-age status of children relative to healthy U.S. reference children. Unlike Pelletier’s work, the studies reviewed by Caulfield’s team contained information about the cause of death, allowing the team to tease out the role of undernutrition in deaths caused by diarrhea, malaria, measles, and pneumonia.

Weight-for-age is the most widely used indicator of child nutritional status in developing countries. Caulfield’s team compared the weight-for-age of children relative to the “international growth reference” established by the National Center for Health Statistics. Children who fall below –2 standard deviations are classified as moderately to severely undernourished (in developing countries, 30–50% of children fall into this category). The team then used a statistical model to relate weight-for-age scores to the death rate.

Overall, the team found having a low weight-for-age score is a leading risk factor for child deaths, accounting for 52.5% worldwide. Among individual diseases studied, undernutrition is responsible for 60.7% of deaths from diarrhea, 57.3% of deaths from malaria, 52.3% of deaths from pneumonia, and 44.8% of deaths from measles.

Moreover, children do not need to be severely undernourished to be at heightened risk of dying if an infectious disease strikes. “Our analysis shows that even children who are small [for their age], but who would not be classified as malnourished based on their weight, are twice as likely to die as children of normal weight,” says Caulfield. “Undernutrition increases the susceptibility to illness and increases the likelihood that an illness will be severe.”

Before Caulfield’s study and the earlier one by Pelletier, experts estimated that undernutrition accounted for no more than 5% of child deaths; cause of death was attributed only to obvious disease symptoms, such as diarrhea or fever. These earlier estimates “did not capture the underlying effect of malnutrition in making a disease more severe,” says Pelletier, who calls undernutrition “the silent killer.”

Public health experts and policy makers historically look to immunizations, drug treatments, and sanitation as ways to prevent child deaths. Programs such as the Millenium Development Goals of the United Nations (which promises to cut the mortality rate of children under age 5 by two-thirds by the year 2015) and vaccination accessibility and research projects funded by the Bill & Melinda Gates Foundation suggest that the international community is committed to improving child health through such means.

But disease treatment and prevention are not enough, says Pelletier; money must also go toward educational and agricultural programs to abate undernutrition. “The impact of undernutrition is not as well appreciated,” agrees Caulfield. Her findings emphasize the need to invest in nutrition programs globally to reduce child deaths.

The new findings are a wakeup call to policy makers about the implications of undernutrition. “The data are there,” says Caulfield, “but we need to translate them for policy makers so that they can understand what it means for a child to weigh less than normal.” In addition to preventing child deaths, correcting undernutrition contributes to quality of life. Even if antibiotics and immunizations keep children alive, “their quality of life is miserable if they’re malnourished,” says Pelletier.

## Figures and Tables

**Figure:**
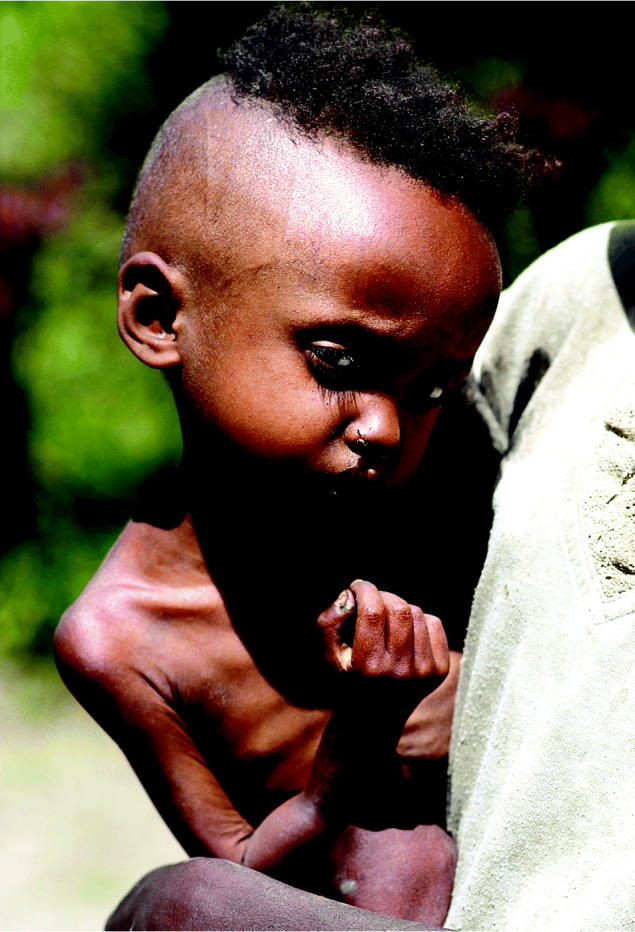
**Hunger persists.** A severely malnourished 4-year-old in Ethiopia is typical of thousands of children around the world whose health and lives are devastated by lack of adequate food.

